# Analogy-Related Information Can Be Accessed by Simple Addition and Subtraction of fMRI Activation Patterns, Without Participants Performing any Analogy Task

**DOI:** 10.1162/nol_a_00045

**Published:** 2022-02-10

**Authors:** Meng-Huan Wu, Andrew J. Anderson, Robert A. Jacobs, Rajeev D. S. Raizada

**Affiliations:** Department of Brain & Cognitive Sciences, University of Rochester, Rochester, New York, USA; Department of Neuroscience, University of Rochester, Rochester, New York, USA; Del Monte Institute for Neuroscience, University of Rochester, Rochester, New York, USA

**Keywords:** fMRI, language, word analogy, word2vec

## Abstract

Analogical reasoning, for example, inferring that *teacher* is to *chalk* as *mechanic* is to *wrench* , plays a fundamental role in human cognition. However, whether brain activity patterns of individual words are encoded in a way that could facilitate analogical reasoning is unclear. Recent advances in computational linguistics have shown that information about analogical problems can be accessed by simple addition and subtraction of word embeddings (e.g., *wrench* = *mechanic* + *chalk* − *teacher*). Critically, this property emerges in artificial neural networks that were not trained to produce analogies but instead were trained to produce general-purpose semantic representations. Here, we test whether such emergent property can be observed in representations in human brains, as well as in artificial neural networks. fMRI activation patterns were recorded while participants viewed isolated words but did not perform analogical reasoning tasks. Analogy relations were constructed from word pairs that were categorically or thematically related, and we tested whether the predicted fMRI pattern calculated with simple arithmetic was more correlated with the pattern of the target word than other words. We observed that the predicted fMRI patterns contain information about not only the identity of the target word but also its category and theme (e.g., teaching-related). In summary, this study demonstrated that information about analogy questions can be reliably accessed with the addition and subtraction of fMRI patterns, and that, similar to word embeddings, this property holds for task-general patterns elicited when participants were not explicitly told to perform analogical reasoning.

## INTRODUCTION

Analogical reasoning is a fundamental component of human cognition. Despite extensive research in psychology (Gentner, 1983), cognitive neuroscience (Chiang et al., 2021; Waltz et al., 1999), and artificial intelligence (Turney, 2006), scientific understanding of the neural bases of analogical problems is limited. Recent advances in computational linguistics have shown that information about analogical problems can be accessed by simple addition and subtraction of word embeddings, which are numeric feature vectors reflecting textual contexts or word co-occurrences (e.g., word2vec; Mikolov et al., 2013). Critically, the ability to access information about analogy problems is emergent in artificial neural networks, because word embeddings are extracted from networks that were trained to produce general-purpose semantic representations, not word analogy relations. While this ability is far from solving analogy problems, it is remarkable that such trivial operations can extract analogy-related information at all.

Given those findings in computational linguistics, it is therefore intriguing to consider whether similar emergent properties can also be observed with biological neural networks, i.e., neural representations in the human brain. In this study, we tested whether the semantic representations of individual words are sufficiently rich such that analogy-related information can be accessed using simple arithmetic operations. Crucially, these patterns were elicited when participants simply read isolated words and were not performing any analogical reasoning task.

**Figure 1.**
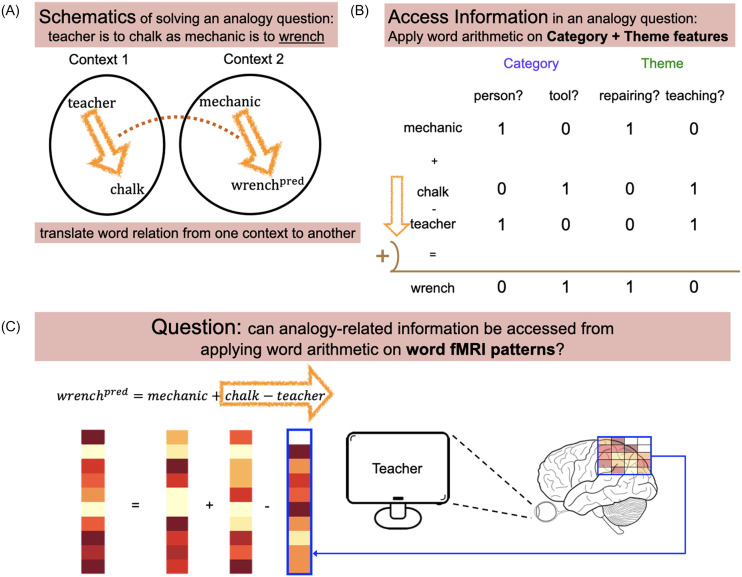
Schematics of the current study. (A) Similar to solving analogy questions, information about word analogies was accessed by translating word relations from one context to another. (B) The categorical and thematic membership of each word can be determined by applying simple word arithmetic operations to such features. (C) We attempted to study the simple yet unanswered question: Can such analogy-related information be accessed from applying addition and subtraction to the fMRI patterns of individual words?

Consider an example analogy question: *teacher* is to *chalk* as *mechanic* is to ( *wrench* ). To solve this question, the relationship between *teacher* and *chalk* must be translated and applied from *teacher* to *mechanic* (Figure 1). We first assume that approximate solutions to analogical questions can be derived geometrically, with word meaning approximated as a numeric feature vector (e.g., word2vec). Then, the relation between *teacher* and *chalk* can be computed by the feature-wise subtraction of *teacher* from *chalk*. Adding the result onto *mechanic* (Figure 1A) will produce a feature vector reasonably close to *wrench* if distinct semantic categories (e.g., tools and people) and contexts (also known as *themes*, e.g., teaching and repairing) are each represented as distinct semantic dimensions (Figure 1B), such that words with different categories and themes occupy distinct positions in the semantic feature space. Following this reasoning, we would anticipate that the segregation of not only themes but semantic categories is also an emergent structural property of word embeddings, which was indeed demonstrated with word2vec (Fu et al., 2014).

There is likewise extensive evidence that information associated with semantic categories and themes is anatomically segregated in the human brain (Kalénine et al., 2009; Lewis et al., 2015; Sachs et al., 2008; Schwartz et al., 2011). These studies have emphasized the role of the anterior temporal lobe (ATL) in representing categories, and the inferior parietal lobe (IPL) in representing themes. This suggests that analogy-related information can be accessed from brain activation patterns (Figure 1C), but only if they are sampled across anatomically separated regions (e.g., IPL and ATL). In contrast, several studies found evidence inconsistent with the segregation perspective; thematic structure has been documented within the ATL (Peelen & Caramazza, 2012), and different semantic categories and themes have been distinguished within individual brain regions (Anderson et al., 2014; Xu et al., 2018). Additionally, word embeddings and other semantic models differentiating categories and themes can explain functional magnetic resonance imaging (fMRI) activation patterns within multiple localized regions of an anatomically distributed semantic network spanning temporal, parietal, and frontal cortex (Anderson et al., 2015, 2019; Carota et al., 2017; Huth et al., 2012, 2016; Mitchell et al., 2008; Pereira et al., 2016, 2018; J. Wang et al., 2017). It is therefore unclear whether analogy-related information can be accessed using fMRI patterns extracted from within individual brain regions, or whether it is necessary to integrate information across the semantic network (e.g., ATL and IPL).

### The Current Study

In this study, we tested two hypotheses. H1: Analogy-related information can be accessed from adding and subtracting task-general fMRI patterns elicited by viewing isolated words (Figure 1C). H2: Analogy-related information can be accessed from adding and subtracting fMRI patterns within individual regions of the brain’s semantic network.

## MATERIALS AND METHODS

### Participants

Sixteen undergraduate students and researchers (age: 23.9 ± 6.8 years; 10 female) participated in the current study. All participants are native English speakers, right-handed, and have normal or corrected-to-normal vision. All participants provided written consent in accordance with the University of Rochester Research Subjects Review Board. Two participants were excluded due to experimental hardware or software failure, and one was excluded for excessive motion artifact (>2 mm in *x*, *y*, *z* dimensions or 2 degrees in pitch, roll, yaw dimensions); therefore, 13 participants were included in the final analysis. We conducted our analysis on 13 participants because this is a standard sample size in other contemporary fMRI studies of isolated word representation that used 9 and 11 participants (Just et al., 2010; Mitchell et al., 2008).

### Stimuli

The 45 words used as stimuli are listed in Table 1. The words were organized into three categories (person/building/tool) and 15 themes. These three categories were selected since it was relatively easy to think up a single theme that would simultaneously apply to all three of these categories (e.g., *doctor-hospital-stethoscope*). We found it difficult to add additional categories that were universally applicable to themes. Stimuli word characteristics such as word length, word frequency, and word imageability ratings are listed in Table 2. Stimuli words are highly imageable overall (*M* = 6.26 on a 7-point scale).

**Table 1.** All 45 words used as stimuli in the current study

	**3 categories (taxonomic)**		
	**Person**	**Place**	**Tool**
**15 themes (thematic)**	doctor	hospital	stethoscope
	soldier	fort	gun
	farmer	barn	plow
	mechanic	garage	wrench
	photographer	studio	camera
	teacher	school	chalk
	chef	restaurant	skillet
	knight	castle	sword
	Eskimo	igloo	harpoon
	bodybuilder	gym	dumbbell
	gardener	greenhouse	mower
	shopper	mall	cart
	judge	court	gavel
	hairdresser	barbershop	trimmer
	chemist	laboratory	beaker

**Table 2.** Characteristics of the 45 stimulus words, organized by each category

**Characteristic**	**Word length mean (*SD*)**	**Word frequency per million mean (*SD*)**	**Imageability mean (*SD*)**
Person	7.40 (2.29)	28.56 (65.85)	6.21 (0.41)
Place	6.47 (2.50)	31.55 (48.67)	6.31 (0.44)
Tool	5.93 (1.94)	7.64 (15.64)	6.28 (0.48)
All categories	6.6 (2.29)	22.58 (48.17)	6.26 (0.43)

*Note*. Word frequency counts are retrieved from the Westbury Lab USENET Corpus (Shaoul & Westbury, 2006). Imageability norms from the available words were retrieved from the Glasgow Norms (Scott et al., 2019).

Words were organized such that an analogy question can be formed by selecting quadruplets associated with two categories and two themes, and finally selecting one of the four words as the target word. For each of the 1,260 distinct analogy questions, we tested whether information about the target word in an analogy question is encoded in the fMRI patterns of the other three words.

### Procedure

Prior to the scanning session, participants filled out the Edinburgh Handedness Inventory form (Veale, 2014) and an adult background form; the forms were to screen whether participants were right-handed and native English speakers respectively. Afterward, they were asked to write down three properties for each of the 45 words on a sheet. This procedure was to ensure that participants were familiar with each word. Note that participants were not provided with the categories and themes throughout the whole experiment and that they were not instructed to perform any task relevant to analogical reasoning.

At the beginning of each run, participants were instructed to form a corresponding mental image upon seeing a word on the screen. For the 45 stimulus word trials, a word selected from Table 1 would appear on the screen for 3 s, followed by a blank screen interstimulus jitter of 3, 4, or 5 s. To engage participants in the task, the stimulus word trials were intermixed with 3 catch trials, where participants saw one of three questions (“Is the previous word a person/building/tool?”) and were instructed to press the left button for answering “No” and the right button for “Yes.” All 45 stimuli appeared exactly once in each run (48 trials), and the trial order was shuffled with the exception that a catch trial could not be the first trial. On average, each run took 324 s (45 word-stimulus trials × 7 s + 3 catch trials × 3 s), which is 162 TRs (where TRs represents repetition time). Participants performed seven runs in total; they completed one practice run on a computer before the scanner session and six runs in the scanner.

### Image Acquisition

Whole-brain images were acquired with a 3-T Siemens MAGNETOM PrismaFit scanner with a 64-channel head coil. A high-resolution structural image was acquired using a T1-weighted MP-RAGE sequence (repetition time = 2,530 ms, echo time = 2.34 ms, flip angle = 7°, field of view = 256 mm, matrix = 256 × 256, 1 × 1 × 1 mm sagittal left-to-right slices). Afterward, each participant was scanned for six functional runs (five runs for one participant). T2*-weighted functional images were acquired with an interleaved echo planar imaging (EPI) pulse sequence (repetition time = 2,000 ms, echo time = 30 ms, flip angle = 70°, field of view = 256 × 256 mm, matrix = 128 × 128, 90 foot-to-head slices, voxel size = 2 × 2 × 2 mm). The first six volumes of each run were discarded to allow the signal to reach steady-state equilibrium.

### fMRI Data Preprocessing

Structural and functional images were processed using statistical parametric mapping (SPM12, v6906; https://www.fil.ion.ucl.ac.uk/spm/software/), and the following preprocessing stages were performed. EPI images were first corrected for head motion and slice time acquisition. Functional images were co-registered to the T1-weighted image. The T1-weighted structural image was segmented into tissue maps, and the resulting deformation field was applied to the functional images to spatially normalize them. No spatial smoothing or voxel spatial clustering was further applied to the images. Preprocessed data were later analyzed with the general linear model. We included 300 regressors in the design matrix: 288 regressors of interest where the onset of each experiment trial (48 trials × 6 runs) was convolved with the standard hemodynamic response function, 6 constant regressors (one for each run) to account for run-to-run signal variations, and 6 motion nuisance regressors. Finally, the blood oxygen level-dependent (BOLD) activation time series for each voxel was fitted to these regressors. The fitted beta coefficients of the three catch trials for each run were discarded and not used for further analyses. The beta coefficients across all runs within each voxel were standardized (i.e., rescaled to have zero mean and unit variance). Note that standardizing the beta coefficients without excluding the target word trials may cause the double-dipping problem (Kriegeskorte et al., 2009) and lead to inflated rankings. To prevent this, we calculated the mean and standard deviation for each voxel on all trials except the target word trials, and we standardized all beta coefficients (including the target word trials) by subtracting out this mean and dividing by this standard deviation. Finally, the fMRI pattern of a word was determined by concatenating the average beta coefficients over the six corresponding trials from the selected voxels.

### Voxel Selection Criterion

For all analyses in the current study, only a subset of the voxels that were relevant to the task was used for the ranking analyses. We chose voxels with the highest stability scores (described below) from all valid voxels in a given region of interest (ROI), i.e., whole brain and the selected brain regions respectively to test H1 and H2. Note that the voxels selected need not be spatially connected, and the selection criterion is not based on the results or significance statistics. For each voxel, we calculated a stability score: a 6-by-6 (or 5-by-5) inter-run correlation matrix was first calculated by taking the Pearson correlation between runs, and the stability score was acquired by averaging the upper triangular part of this matrix (see Mitchell et al., 2008 for similar methods). Intuitively, this criterion selected voxels that were consistently activated by the word stimuli across multiple runs. To ensure that the analyses aren’t sensitive to the voxel selection criterion, we selected a wide range number of stable voxels (i.e., from 100 to 6,400 voxels) for the first analyses. For the ROI analyses, the most stable 100 voxels in each ROI were selected. To exclude the fMRI activation patterns of the target word from the calculation of the predicted fMRI pattern (described in the Experimental Design and Statistical Analysis section), for every analogy question we calculated the voxel stability scores without the target word.

### Experimental Design and Statistical Analysis

**Figure 2.**
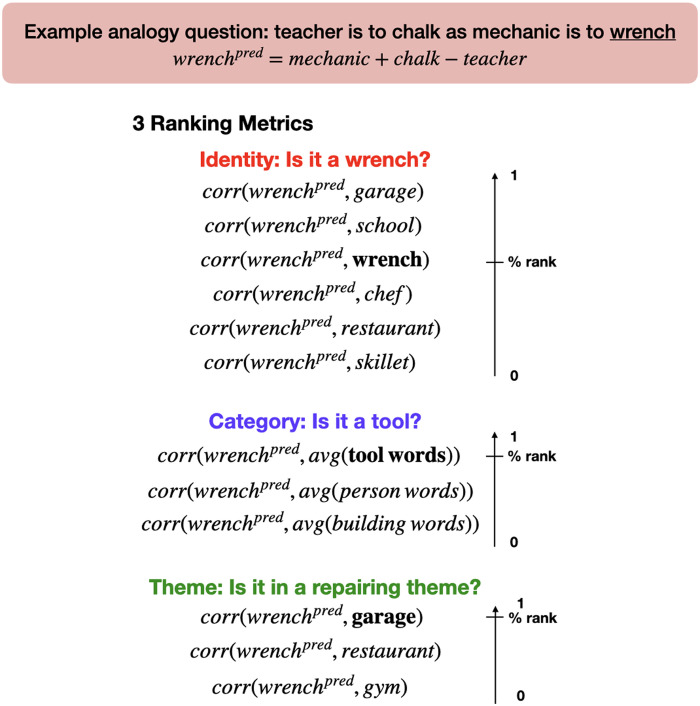
Description of the ranking metrics. Top: The example analogy question and the arithmetic operations we applied to create the predicted fMRI pattern (i.e., *wrench* ^*pred*^). The target word (i.e., *wrench*) is underlined. Bottom: The three main ranking metrics. For each metric, the Pearson correlation coefficient between *wrench* ^*pred*^ and the target pattern (in bold font) was ranked against the correlation between *wrench* ^*pred*^ and all other candidate words. The rank was scaled to [0, 1] and averaged across all four target words in all possible analogy questions. In the identity metric the target pattern was the fMRI pattern of the target word (i.e., *wrench*), and all other words in the stimuli list, except the four words in the analogy question, were candidate words. In the category metric, category templates, which were the averaged fMRI patterns across all remaining words in each category, were used instead of the fMRI patterns of individual words. The target category template was the category that the word actually belonged to, and the two other templates served as candidates. In the theme metric, the target pattern was the word in the same theme and the final unused category (i.e., *garage*), and all other words in the unused category (i.e., building words) were candidate words.

To test the first hypothesis (H1) of whether analogy-related information can be accessed from adding and subtracting fMRI activation patterns associated with individual words, we devised five ranking metrics (Figure 2) to examine whether information about the identity, category, and theme of a word can be accessed. Intuitively, if the predicted fMRI pattern contains information about certain categories, it should be more similar to the word patterns in that category than others (and the ranking would be higher). Note that ranking metrics instead of raw distance metrics were used since we are predominantly interested in whether the distance between the predicted pattern and the ground truth word is shorter than that for other words, not the magnitude of the distance itself. Furthermore, the various analyses performed with different numbers of voxels and brain regions can be compared directly with the ranking metrics.

Each test metric was computed in three steps: (1) construct the predicted fMRI pattern, (2) calculate the correlations between the predicted pattern and that of candidate words, and (3) rank the correlations. To illustrate how the ranking metrics were calculated, we used the following example analogy: *teacher* is to *chalk* as *mechanic* is to ( *wrench*), where *wrench* is the target word. Similar to the arithmetic operations used in word2vec (Mikolov et al., 2013), the predicted fMRI pattern of the target word was simply calculated as the addition/subtraction of other word fMRI patterns in the question:

$${\text{wrench}}^{\text{pred}}=\text{mechanic}+\text{chalk}-\text{teacher}$$ (1)

The ranking for this target word was calculated as:

1. Identity metric: We tested whether the predicted fMRI pattern is closer to the true word *wrench* compared to other words. Specifically, we calculated and ranked the Pearson correlation coefficients between *wrench* ^*pred*^ and the fMRI patterns of all other 42 words in the stimuli list (the three words used to create *wrench* ^*pred*^, i.e., *mechanic*, *chalk*, and *teacher*, were excluded). Intuitively, the ranking of the corresponding correlation coefficient between *wrench* ^*pred*^ and the ground truth pattern *wrench* represents how close the predicted pattern is to the true word compared to other words. Finally, the ranking (value between 1 and 42) is linearly scaled to the 0 ∼ 1 range to facilitate comparison between different ranking metrics, which can have different numbers of candidate words.
2. Category metric: To test which category (i.e., person/building/tool) *wrench* ^*pred*^ belonged to, we tested whether it was more similar to a template canonical representation of tool words than person words and building words. First, the template of each category was calculated by taking the voxel-wise mean of the fMRI patterns of all 13 remaining words in that category (e.g., the tool template was the average of the tool words that remained after the two tool words in the analogy question were excluded). Second, we ranked the Pearson correlation coefficients between *wrench* ^*pred*^ and the three category templates. Finally, the rank associated with the true category template was scaled to the 0 ∼ 1 range.
3. Theme metric: To test which theme (e.g., education, shopping, etc.) *wrench* ^*pred*^ belonged to, we first gathered all words belonging to the last category unused in the analogy question (e.g., building words in the example question), and tested whether *wrench* ^*pred*^ was closer to the word in the same theme (i.e., *garage*) compared to the other 13 words (e.g., *mall*, *court*). Similarly to other metrics, we extracted the rank of the correlation between *wrench* ^*pred*^ and *garage* and scaled it to the 0 ∼ 1 range.
4. Identity (within-category) metric: This metric serves to eliminate the confound that the identity metric can be significant when the fMRI patterns only encode category information. Unlike the Identity metric, the Pearson correlation coefficients were only calculated between *wrench* ^*pred*^ and the fMRI patterns of all other words in the true category. Similarly, the rank of correlation between *wrench* ^*pred*^ and the ground truth pattern *wrench* is scaled to the 0 ∼ 1 range.
5. Identity (close foil) metric: In standard analogical reasoning paradigms, a target word is usually probed against a close foil word that answers the analogy question partially; for instance, *garage* is a close foil word as it has the same theme as but a different category from the target word. We tested whether *wrench* ^*pred*^ is closer to the ground truth pattern *wrench* than the fMRI pattern of a close foil word, which was selected in the current study to be the unused word in the same theme (e.g., *garage*).

The procedure above describes how the five ranking metrics were calculated in one analogy question. To assess whether analogy-related information can be extracted from fMRI patterns in a ROI, we performed a group-level statistical analysis. To facilitate this, in each of the five tests, we reduced each individual’s rank scores to a single summary metric, by averaging ranks (within each participant) across all analogy questions. We then performed a group-level test of whether the mean rankings were greater than data-driven estimates of chance-level computed for each participant. To determine the chance level, we first randomly shuffled the correspondence between word labels and their fMRI patterns, ran the ranking metrics with the shuffled data, and calculated the average ranking across all analogy questions. This permutation process was repeated 1,000 times for each participant, each ROI, and each metric, and we aggregated the 1,000 results to get the null mean. Finally, for each ROI and each metric, we performed a one-sided paired *t* test between the real ranking metrics (13 points, one from each participant) and the null mean from each participant (also 13 points). Note that since the rankings were scaled to the 0 ∼ 1 range, the null means were close to the theoretical chance level of 0.5 (*M* = 0.4999, *SD* = 0.001). The paired *t* test is preferred over the standard one since we can empirically estimate the chance level instead of assuming it to be 0.5, and the two tests yield quantitatively similar results. For the ROI-level analysis, *p* values associated with multiple ROIs were corrected for multiple comparisons using the false discovery rate method (Benjamini & Hochberg, 1995). In light of recent concerns over statistical testing in multivariate pattern analyses (Allefeld et al., 2016), it is important to note that the current rank measure can be any value between 0 and 1 and should be distinguished from information-like measures such as classification accuracy.

Finally, to claim that analogy-related information can be accessed from the fMRI patterns, we consider that not only the identity metric but also the category and theme metrics must yield test statistics that are significantly above chance. This is because above-chance ranks in the identity metric do not automatically entail that information reflecting *both* the theme and category of the word is represented in fMRI. Thus, if only the category but not the theme is encoded in fMRI we might end up with computations (in place of Equation 1) that reduce to:

$${\text{wrench}}^{\text{pred}}=\text{person}+\text{tool}-\text{person}=\text{tool}$$ (2)

Since *wrench* is a tool, the predicted fMRI pattern would be ranked higher than the other person and building words in the identity metric (rank = 0.66) and therefore above the 0.5 chance-level. One way to eliminate this possibility is to make sure that both the category and the theme metrics are significantly above chance, and therefore the fMRI patterns encode both category and theme information. Alternatively, a significant identity (within-category) metric can also suggest that the word identity information can be accessed even among stimulus words in the same category. In sum, we consider that the identity, category, and theme ranking metrics must all be significantly above chance to claim evidence of successfully accessing analogy-related information.

### Regions of Interests Analysis

**Figure 3.**
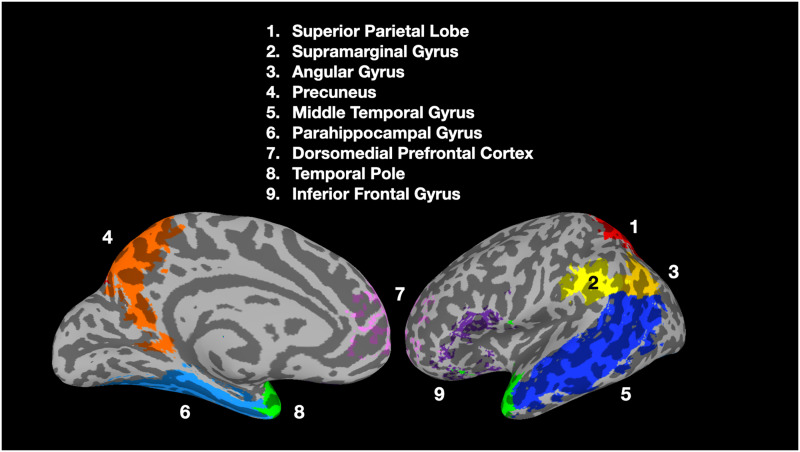
Nine prespecified semantic related ROIs. Colors denote different ROIs and not the significance of results.

To test the second hypothesis (H2) of whether analogy-related information can be accessed within particular brain regions (as opposed to across brain regions), nine anatomically prespecified ROIs (Figure 3) were selected. These ROIs were selected for their roles in various semantic processing and analogical reasoning (Carota et al., 2017; Chiang et al., 2021; Hobeika et al., 2016) tasks, such as processing semantic category relations (Schwartz et al., 2011) and representing semantic similarity among concepts (Patterson et al., 2007). The parietal lobe ROIs were created using the automated anatomical labeling atlas (Rolls et al., 2015; Tzourio-Mazoyer et al., 2002). The inferior frontal gyrus and the dorsomedial prefrontal cortex were created from the union of BA 44, 45, and 47 and the union of BA 2 and 10 respectively, where the regions were collected from the Wake Forest University (WFU) Pickatlas toolbox (https://school.wakehealth.edu/Research/Labs). While these ROIs were defined on the left hemisphere due to the left hemispheric dominance observed in the aforementioned studies, we also conducted a secondary analysis using the right hemispheric homologs of these ROIs in light of a reviewer’s query (see the results in Supp. Figure 2; Supporting information can be found online at https://doi.org/10.1162/nol_a_00045). We note that this is a post-hoc analysis since the ROIs were not selected prior to the experiment.

## RESULTS

### Task-General fMRI Patterns of Individual Words Contain Information About Analogy Relations

**Figure 4.**
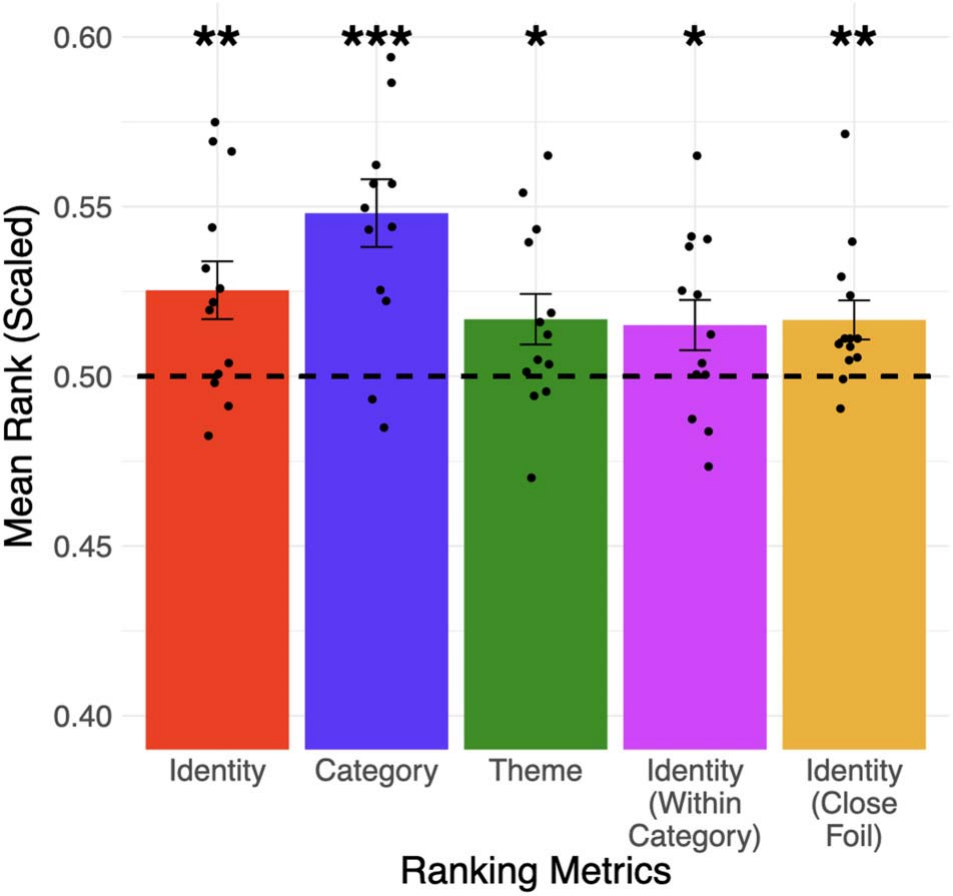
Information about the identity (e.g., *wrench* vs. 41 other words), category (e.g., tool vs. two other categories), theme (e.g., repairing vs. all 13 other themes) of a word can be accessed from the addition and subtraction of fMRI patterns of the three other words in an analogy question. The identity of the target word can also be distinguished from words in its category (4th column) and a foil word from its theme (5th column). Error bars represent the standard error of the mean across participants (* *p* < 0.05; ** *p* < 0.01; *** *p* < 0.001). The dotted line represented chance-level performance (mean rank = 0.5) derived from the participant-level permutation process. Black dots represent the mean rank of each participant. This figure only displays results from fMRI patterns where 100 voxels were selected according to the voxel selection procedure (see Materials and Methods), and they do not need to be spatially connected; see Supp. Table 1 for the full results.

Can analogy-related information be accessed from the addition and subtraction of fMRI activation patterns? We tested five ranking metrics (Figure 2) using fMRI patterns extracted from the whole brain. We found that the identity (paired *t* test, mean = 0.525, *SD* = 0.03, *p* = 0.006) and category (paired *t* test, mean = 0.548, *SD* = 0.04, *p* < 0.001) metrics are significantly higher than chance at the group-level (i.e., across all participants) (Figure 4), and both effects are stable across a wide range of voxel selection criteria (up to 3,200 voxels, see Supp. Table 1). The theme metric is also significantly above chance (paired *t* test, mean = 0.517, *SD* = 0.03, *p* = 0.02), but only when up to 200 voxels are selected. We also found significant results (paired *t* test, mean = 0.515, *SD* = 0.03, *p* = 0.03) with the identity (within-category) metric (i.e., we tested whether the predicted pattern is closer to the actual patterns compared to other words in the same category); this eliminated the confound that the identity metric can be significant when the fMRI patterns only encode category information. Finally, we drew inspiration from common analogical reasoning paradigms and found that the fMRI patterns of the actual word can be reliably distinguished from that of a close foil (i.e., the identity (close-foil) metric is significant: paired *t* test, mean = 0.517, *SD* = 0.02, *p* = 0.007). Overall, the results support our first hypothesis (H1) that analogy-related information from individual words can be reliably accessed, but further analysis of the ROI-level is required.

### Word Categories and Themes Were Jointly Represented in the Parietal Lobe

We further investigated whether analogy-related information can be accessed within single brain regions (as opposed to extracting fMRI patterns across multiple regions). While previous large-scale neuroimaging studies suggested that semantic properties were widely distributed across the whole brain (Huth et al., 2012, 2016), other studies argued that category and theme information were localized in distinct brain regions (Kalénine et al., 2009; Sachs et al., 2008; Schwartz et al., 2011). It is therefore of scientific interest to examine whether analogy-related information can be accessed within a single region (i.e., both category and theme information were represented). As a result, we ran the five ranking metrics on nine prespecified ROIs in the left hemisphere (Figure 3); these hypothesis-driven ROIs were selected based on their role in processing semantic and analogical relations (Carota et al., 2017; Chiang et al., 2021; Schwartz et al., 2011), representing semantic similarity among concepts (Patterson et al., 2007), and as a transmodal hub to generalize across various modality-specific semantic information (Lambon Ralph et al., 2017).

**Figure 5.**
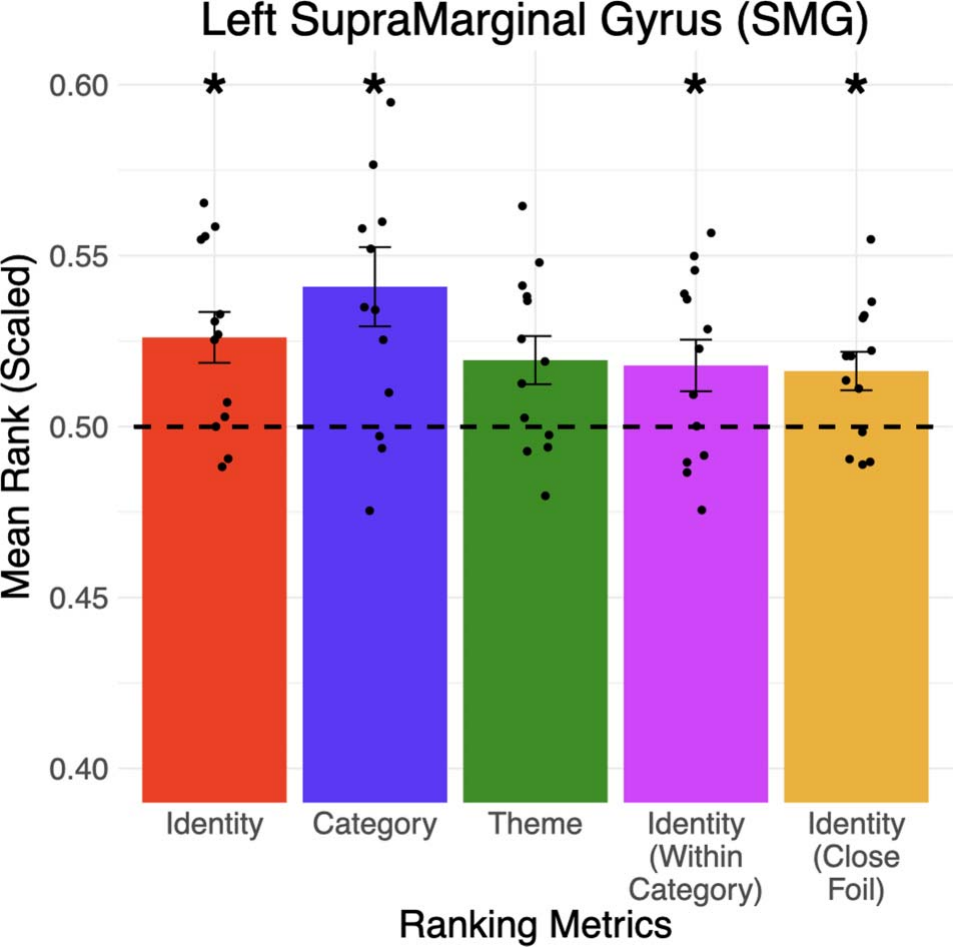
Information about the identity (e.g., *wrench* vs. 41 other words), category (e.g., tool vs. two other categories), and theme (e.g., repairing vs. all 13 other themes) of a word can be accessed from the addition and subtraction of fMRI patterns in the left supramarginal gyrus. The identity of the target word can also be distinguished within its category (4th column) and from a close foil word in its theme (5th column). Error bars represented the standard error of the mean across participants (* *p* < 0.05). The dotted line represented chance-level performance (mean rank = 0.5). Black dots represent the mean rank of each participant.

As shown in Table 3, among the nine ROIs, the identity ranking of the left superior parietal lobe, supramarginal gyrus, angular gyrus, precuneus, and middle temporal gyrus were significantly above chance. The category metric was significantly above chance in most ROIs except the dorsomedial prefrontal cortex and temporal pole. Regarding the theme metric, while none of the ROIs are significant after multiple correction, the left supramarginal gyrus (see Figure 5) and angular gyrus were borderline (*p* = 0.05 post correction). Prompted by a reviewer, we also conducted an analysis of the eight right-hemispheric homologs of the above left-hemisphere ROIs (see Supp. Figure 2). The findings suggested that analogy-related information was encoded more weakly in the right hemisphere, with only category and identity metrics significantly above chance in the right supramarginal gyrus, angular gyrus, and precuneus. Overall, the results hinted toward our second hypothesis (H2) that the fMRI patterns of individual words in the semantic network regions encode analogy-related information, similar to the distributed word-embedding models (Mikolov et al., 2013).

**Table 3.** The mean rank (*M*) and corrected *p* values of five ranking metrics in the nine prespecified semantic-related ROIs (all in the left hemisphere)

**ROIs (left)**	**Identity**		**Category**		**Theme**		**Identity (within category)**		**Identity (close foil)**	
	***M***	***p***	***M***	***p***	***M***	***p***	***M***	***p***	***M***	***p***
Superior parietal lobe	**0.521**	0.03*	**0.538**	0.02*	0.516	0.08	0.513	0.11	0.508	0.18
Supramarginal gyrus	**0.526**	0.003**	**0.541**	0.01*	0.519	0.05 (0.006)	0.518	0.05	**0.516**	0.03*
Angular gyrus	**0.522**	0.02*	**0.530**	0.003**	0.518	0.05 (0.01)	0.517	0.05	**0.506**	0.03*
Precuneus	**0.525**	0.03*	**0.532**	0.02*	0.519	0.08	0.519	0.11	0.510	0.07
Middle temporal gyrus	**0.521**	0.03*	**0.535**	0.03*	0.511	0.08	0.512	0.12	**0.514**	0.03*
Parahippocampal gyrus	0.516	0.16	**0.526**	0.003**	0.511	0.32	0.511	0.36	**0.510**	0.03*
Dorsomedial prefrontal cortex	0.505	0.26	0.528	0.07	0.501	0.43	0.500	0.43	0.505	0.26
Temporal pole	0.513	0.22	0.522	0.29	0.506	0.43	0.507	0.38	**0.511**	0.04*
Inferior frontal gyrus	0.511	0.17	**0.521**	0.05*	0.507	0.32	0.506	0.36	0.503	0.18

*Note*. Uncorrected *p* values are shown in parentheses. Significant metrics are marked in bold. While category information seems to be represented in widespread regions, theme information is only present in the parietal lobe regions.

## DISCUSSION

In the current study, we first tested the hypothesis (H1) of whether analogy-related information can be accessed from adding and subtracting fMRI activation patterns elicited by viewing individual words. We demonstrated that information about the identity, category, and theme of the target word can be accessed from the fMRI activation patterns. Critically, it is important to note that participants were only viewing individual words in the experiment without knowing the structure of the stimuli list, and they were not asked to perform analogical reasoning of any sort. This implies that like word embeddings (e.g., word2vec) extracted from artificial neural networks, task-general semantic representations of individual words in the human brain are sufficiently rich that simple arithmetic operations can be directly applied to them to access analogy-related information, and that explicit analogical reasoning tasks are not necessary to activate them.

We further tested the second hypothesis (H2) of whether analogy-related information can be accessed within individual semantic network regions, or whether it is necessary to integrate voxels from multiple areas. Our results demonstrated that while the category of a target word could be accessed from fMRI patterns extracted from multiple parietal, temporal, and frontal regions, theme information can only be accessed within the left parietal lobe region. This is consistent with previous studies demonstrating that first-order analogical relations were present in the superior parietal cortex (Chiang et al., 2021). The finding that category information was encoded in more regions may have arisen from the category test being more powerful, or the particular categories and themes comprising the stimuli. While this result was consistent with previous studies demonstrating that the ATL encoded category relations and the IPL encoded thematic relations (Schwartz et al., 2011), it newly reveals that the multiple parietal lobe regions also contained category information. However, since tool was one of the three categories used in the current study and task-general tool processing is associated with left IPL activation (Ishibashi et al., 2016), it is not altogether surprising that the IPL could distinguish tool words from other categories. Overall, the results suggest that the intrinsic organization of semantic representations within an individual brain region contains analogy-related information locally (by addition and subtraction).

The ranking accuracies in the current study were not high, although they were nonetheless significantly above chance. This could have been for several reasons. First, the predicted fMRI patterns for the target word were calculated based on integrating information across fMRI activation patterns corresponding to only three words. This is a very small training set in comparison to contemporary studies that have not only used more words to correlate with fMRI data but also applied regression-based approaches to map fMRI patterns to a semantic model (e.g., Mitchell et al., 2008 used 58 words as a training set). Indeed, the current approach that tests the hypotheses without parameter fitting resonates with our previous approach (Anderson et al., 2016) to sidestep model overfitting problems and combine the strength of both encoding analysis and representational similarity analysis (Kriegeskorte et al., 2008). Second, while previous studies have found better model performance with richer stimuli formats such as words associated with line drawings (Mitchell et al., 2008), images, sentences, and word clouds (Pereira et al., 2018), we showed participants individual words only. While this paradigm has seen success in previous studies (Just et al., 2010), recent studies comparing fMRI word decoding performance between different stimuli formats hinted that presenting stimuli in multiple modalities may activate a broader network of brain regions and lead to higher accuracies (Pereira et al., 2018; S. Wang et al., 2020).

It is critical to restate that the fMRI patterns were elicited when participants were not performing an analogical reasoning task, since this study aimed to examine whether analogy-related information could be accessed from task-general fMRI patterns. If, contrary to what was actually done, we had instructed participants to perform an analogical reasoning task, then we would have risked activation specific to the analogy task obscuring and/or modifying the task-general representations of isolated words we sought to test. For instance, in a typical analogical reasoning task, participants might see two pairs of words and then determine whether the relations between the two pairs were identical or not. While it has been demonstrated that different types of relations (e.g., similarity and contrast) can be decoded from semantic network regions (Chiang et al., 2021), it remains unclear how they can be calculated from the task-general fMRI patterns of their component words. Since the current study demonstrated that task-general fMRI patterns were intrinsically organized and contain analogy-related information, it serves as initial evidence that a potential mechanism to calculate such relations is to simply add and subtract fMRI patterns of individual words. Future studies are necessary to elucidate the relationship between task-general fMRI patterns and the representations elicited during explicit analogical reasoning tasks, and whether the former are recruited in the construction of the latter.

The current study complements previous fMRI studies of analogical reasoning (Chiang et al., 2021) in the following respects. First, whereas Chiang et al. (2021) examined the neural computations underlying actual analogical reasoning, our study investigated: (1) participants who had no knowledge that the experiment was related to analogical reasoning; (2) fMRI activation elicited by viewing isolated words; and (3) whether fMRI activation patterns associated with isolated words were inherently structured to reflect their analogical relationship. Second, Chiang et al. (2021) demonstrated that fMRI activation patterns elicited during analogical reasoning were correlated with patterns of features that explicitly estimated how pairs of word2vec vectors were related, as synonyms, antonyms, cause-effect, and so on (i.e., BART; Lu et al., 2012). Word2vec was not used in the current study; instead, the simple arithmetic operations of addition and subtraction used to access analogy-related information were directly applied to combine fMRI activation patterns associated with individual words.

The current study has several limitations. First, this study only considered concrete nouns that were analogically related to each other categorically or thematically. While previous studies have shed light on how abstract concepts were structured in the human brain (Anderson et al., 2014, 2017; Pereira et al., 2018; Vargas & Just, 2019; X. Wang et al., 2018), future work is needed to investigate whether the semantic organization of abstract nouns or adjectives in the brain is organized such that analogy-related information can be similarly accessed. Second, the tool words in the stimuli list have lower word frequency compared to words in other categories, and less-frequent words might elicit higher activation values in brain regions such as the left inferior frontal gyrus (Schuster et al., 2016). However, at the same time, the tool words were not obscure (e.g., *gun*, *wrench*, *skillet*) and word frequency alone could not account for all of the ranking metrics, in particular the theme metric that compares words within the same category. Third, the categories chosen are quite distinct, and it is challenging to eliminate every confounder that varies between any two categories; for instance, living vs. nonliving can also differentiate person words from the others. Again, these confounders cannot drive the significant results in the theme metric. Further studies will be necessary to test whether the current results can be generalized to finer-grained categories. Finally, our word stimuli varied in length, because it was beyond our ability to devise a suitable list of words with the same length. While word length is an important factor in semantic processing and reading (Just et al., 2010; Schuster et al., 2016), its influence has mostly been observed in the occipital pole and lingual/fusiform gyri (Just et al., 2010) and is unlikely to have had a substantial influence on the results of the current study (e.g., Figure 5).

### Conclusions

In sum, the current study has demonstrated that analogy-related information can be accessed by applying addition and subtraction to fMRI patterns elicited by reading isolated words. It has further identified regions of the brain’s semantic network that represent semantic categories and themes and revealed evidence that word representations within the left parietal lobe region could sufficiently contain such information. For the broader literature investigating how humans perform analogical reasoning tasks, the current study has provided initial evidence that the relation between two words can be approximated by applying simple addition and subtraction on task-agnostic fMRI patterns of individual words.

## ACKNOWLEDGMENTS

We thank Carol Jew for insightful comments and discussions. We also thank Dave Kleinschmidt for providing analysis code. This work was supported by a Google Faculty Award, NSF CAREER award 1652127, and NSF research grant DRL-1561335.

## FUNDING INFORMATION

Rajeev Raizada, Google (https://dx.doi.org/10.13039/100006785). Rajeev Raizada, National Science Foundation (https://dx.doi.org/10.13039/100000001), Award ID: 1652127. Robert A. Jacobs, National Science Foundation (https://dx.doi.org/10.13039/100000001), Award ID: DRL-1561335.

## AUTHOR CONTRIBUTIONS

**Meng-Huan Wu**: Conceptualization: Equal; Data curation: Lead; Formal analysis: Lead; Methodology: Lead; Software: Lead; Visualization: Lead; Writing – original draft: Lead. **Andrew J. Anderson**: Conceptualization: Equal; Data curation: Supporting; Supervision: Supporting; Writing – review & editing: Supporting. **Robert A. Jacobs**: Supervision: Lead; Writing – review & editing: Supporting. **Rajeev D. S. Raizada**: Supervision: Lead; Writing – review & editing: Supporting.

## TECHNICAL TERM

Functional magnetic resonance imaging (fMRI): A technique that measures brain activity and is used to study brain function.
