# Supplementary material for: Analogy-Related Information Can Be Accessed by Simple Addition and Subtraction of fMRI Activation Patterns, Without Participants Performing any Analogy Task
Source: Neurobiol Lang (Camb). 2022 Feb 10;3(1):1–17. doi: 10.1162/nol_a_00045 (PMC10158578; doi:10.1162/nol_a_00045)
Supplement: Supplementary file 1 [file nol-3-1-1-s001.docx]

Supplementary Materials

| Voxels selected | Identity | Category | Theme | Identity (Within Category) | Identity (Close Foil) |
| --- | --- | --- | --- | --- | --- |
| 100 | 0.525 ** | 0.548 *** | 0.517 * | 0.515 * | 0.517 ** |
| 200 | 0.524 * | 0.545 *** | 0.515 * | 0.514 | 0.518 ** |
| 400 | 0.524 * | 0.540 ** | 0.515 | 0.515 | 0.515 ** |
| 800 | 0.523 * | 0.539 ** | 0.516 | 0.515 | 0.512 ** |
| 1600 | 0.521 * | 0.538 *** | 0.514 | 0.514 | 0.507 |
| 3200 | 0.520 * | 0.534 ** | 0.513 | 0.512 | 0.508 * |
| 6400 | 0.517 | 0.530 ** | 0.511 | 0.511 | 0.504 |

Supp. Table 1: The ranking of the five ranking metrics (averaged across all analogy questions) across a wide range of voxel selection criteria in the first analysis. Group-level statistical analysis results are shown with * (*: p < 0.05, **: p < 0.01, ***: p < 0.001).

|  | Identity | | Category | | Theme | | Identity (Within Category) | | Identity  (Close Foil) | |
| --- | --- | --- | --- | --- | --- | --- | --- | --- | --- | --- |
| ROIs (right) | M | P | M | p | M | p | M | p | M | p |
| superior parietal lobe | 0.511 | 0.14 | 0.524 | 0.12 | 0.509 | 0.21 | 0.505 | 0.28 | 0.502 | 0.30 |
| supramarginal gyrus | 0.511 | 0.14 | **0.526** | **0.01*** | 0.508 | 0.21 | 0.508 | 0.27 | **0.510** | **0.05*** |
| angular gyrus | 0.516 | 0.06 | **0.539** | **0.01*** | 0.508 | 0.21 | 0.505 | 0.28 | **0.514** | **0.04*** |
| precuneus | 0.519 | 0.06 | **0.532** | **0.01*** | 0.511 | 0.21 | 0.511 | 0.27 | **0.513** | **0.04*** |
| middle temporal gyrus | 0.511 | 0.14 | 0.518 | 0.12 | 0.507 | 0.21 | 0.508 | 0.27 | 0.503 | 0.29 |
| parahippocampal gyrus | 0.512 | 0.14 | 0.513 | 0.15 | 0.508 | 0.21 | 0.509 | 0.27 | 0.503 | 0.28 |
| dorsomedial prefrontal cortex | 0.507 | 0.26 | 0.507 | 0.31 | 0.509 | 0.21 | 0.507 | 0.28 | 0.497 | 0.71 |
| temporal pole | 0.510 | 0.18 | 0.509 | 0.29 | 0.509 | 0.21 | 0.509 | 0.27 | 0.502 | 0.30 |

Supp. Table 2. The mean rank (M) and corrected p-values (across 8 ROIs) of five ranking metrics in the 8 right hemisphere ROIs. Significant metrics were marked in bold.
